# Comparison between calcium hydroxide mixtures and mineral trioxide aggregate in primary teeth pulpotomy: a randomized controlled trial

**DOI:** 10.1590/1678-7757-2018-0030

**Published:** 2019-05-20

**Authors:** Lidiane Lucas Costa e Silva, Leopoldo Cosme-Silva, Vivien Thiemy Sakai, Camila Soares Lopes, Ana Paula Pereira da Silveira, Rafael Tobias Moretti, João Eduardo Gomes-Filho, Thais Marchini Oliveira, Ana Beatriz da Silveira Moretti

**Affiliations:** 1 Universidade Federal de Alfenas (UNIFAL), Faculdade de Odontologia, Departamento de Clínica e Cirurgia, Alfenas, Minas Gerais, Brasil.; 2 Universidade Estadual Paulista (UNESP), Faculdade de Odontologia de Araçatuba, Departamento de Dentística Restauradora - Endodontia, Araçatuba, São Paulo, Brasil.; 3 Universidade de São Paulo, Faculdade de Odontologia de Bauru, Departamento de Odontopediatria, Ortodontia e Saúde Coletiva, Bauru, São Paulo, Brasil.

**Keywords:** Calcium hydroxide, Deciduous tooth, Mineral trioxide aggregate, Pulpotomy, Randomized trial

## Abstract

**Objectives:**

To evaluate the effect of calcium hydroxide (CH) associated with two different vehicles as a capping material for pulp tissue in primary molars, compared with mineral trioxide aggregate (MTA).

**Methodology:**

Forty-five primary mandibular molars with dental caries were treated by conventional pulpotomy using one of the following materials: MTA only (MTA group), CH with saline (CH+saline group) and CH with polyethylene glycol (CH+PEG group) (15 teeth/group). Clinical and periapical radiographic examinations of the pulpotomized teeth were performed 3, 6, and 12 months after treatment. Data were tested by chi-squared analysis and a multiple comparison post-test.

**Results:**

The MTA group showed both clinical and radiographic treatment success in 14/14 teeth (100%), at all follow-up appointments. By clinical evaluation, no teeth in the CH+saline and CH+PEG groups had signs of mobility, fistula, swelling or inflammation of the surrounding gingival tissue. However, in the CH+saline group, radiographic analysis detected internal resorption in up to 9/15 teeth (67%), and inter-radicular bone resorption and furcation radiolucency in up to 5/15 teeth (36%), from 3 to 12 months of follow-up. In the CH+PEG group, 2/11 teeth (18%) had internal resorption and 1/11 teeth (9%) presented bone resorption and furcation radiolucency at all follow-up appointments.

**Conclusion:**

CH with PEG performed better than CH with saline as capping material for pulpotomy of primary teeth. However, both combinations yielded clinical and radiographic results inferior to those of MTA alone.

## Introduction

Pulpotomy is a vital pulp therapy for deciduous teeth in which the coronal pulp tissue is surgically removed and a suitable material is placed on the remaining radicular pulp to protect it from further injury.^1^ ^,^ ^2^ ^,^ ^3^ ^,^ ^4^ ^,^ ^5^ Pulpotomy aims to retain a functional tooth with a vital radicular pulp in the oral cavity until its exfoliation.^1^ ^,^ ^2^ ^,^ ^6^ ^,^ ^7^ ^,^ ^8^

Although formocresol was considered the gold standard for pulpotomies in primary teeth, its toxicity and carcinogenic potential have emerged as serious limitations of this capping material.^9^ Numerous studies have investigated the mechanism of action, clinical indication, advantages and disadvantages of different capping materials, but no consensus has yet been reached on the ideal pulpotomy material for the treatment of primary teeth.^1^ ^,^ ^4^ ^,^ ^6^ ^,^ ^8^ ^,^ ^10^ ^,^ ^11^

Calcium hidroxide (CH) has been indicated as the most appropriate material in many clinical situations aiming to promote healing. However, the results obtained from pulpotomy using CH-based materials were inconclusive, as long-term clinical trials revealed an increase in failure rates along the follow-up appointments.^6^ ^,^ ^12^ The success rate of CH as a pulpotomy material in primary teeth is poor in comparison to that observed in permanent teeth. The caustic action of the high-pH formulations of CH reduces the size of the underlying dental pulp by up to 0.7 mm.^13^ In addition to the widespread clinical use of CH, studies have tested various CH formulations and mixtures of CH powder with different substances in an attempt to improve CH performance.^12^ ^,^ ^14^

Typically, CH dressing is prepared with either an aqueous (distilled water, saline, anesthetic solution) or viscous (polyethylene glycol, propylene glycol, methylcellulose, glycerin) vehicle.^14^ ^,^ ^15^ When in direct contact with tissues, CH paste prepared with an aqueous vehicle rapidly dissociates into calcium (Ca^+2^) and hydroxyl (OH^−^) ions, promoting high solubility and becoming easily resorbed by macrophages. In contrast, when CH is mixed with a viscous vehicle, its dissociation is slower, probably because of the high molecular weight of the vehicle, thus minimizing the dispersion of CH into the underlying tissue and maintaining the paste in the desired place for longer.^14^

MTA is considered the gold standard material for pulpotomy of primary teeth.^3^ ^,^ ^5^ ^,^ ^7^ ^,^ ^9^ Its indication is based on its adequate physiochemical and biological properties such as good sealing ability, hydroxyapatite formation and favorable biocompatibility.^8^ ^,^ ^16^ ^,^ ^17^ MTA induces hard tissue formation when used for pulp capping, pulpotomy, perforated furcations, root canal filling and root-end fillings in animals;^12^ ^,^ ^15^ ^,^ ^17^ however, it is costly and causes dental pigmentation.^11^ ^,^ ^18^ ^,^ ^19^

Considering the scarcity of randomized controlled trials testing CH associated with different vehicles as a dressing agent for primary teeth, this study aimed to evaluate the effect of CH prepared with either an aqueous (saline) or a viscous (polyethylene glycol) vehicle as a capping material for the pulpotomy of primary molars. Of note, the effects of CH mixtures were compared with those of MTA, which represents the current standard of treatment for primary teeth pulpotomy.

## Methodology

### Participants and ethics

This prospective and randomized clinical trial was registered in the ISRCTN (registration number: ISRCTN51317677) and approved by the Ethics Committee of the School of Dentistry (Federal University of Alfenas; protocol #15304613.1.0000.5142). The trial was conducted from July 2013 to July 2015 at the School of Dentistry from the Federal University of Alfenas, involving 39 children on whom 45 pulpotomies were performed. The parents or guardians of the children involved received pertinent information regarding the study and provided written informed consent for their children's participation in the trial. No monetary compensation for their participation was provided. All participants were blinded to the treatment group to which they were allocated.

The inclusion criteria used in this study were: mandibular primary first or second molar of children aged between 5 and 8 years and belonging to the American Society of Anesthesiologists (ASA) physical status category I; no history of pain; adequate clinical macroscopic appearance of the dental pulp (normal consistency, bright red color and cut resistance); no clinical or radiographic evidence of pulp degeneration, such as excessive bleeding, internal root resorption, inter-radicular and/or furcal bone destruction; and no more than one third of physiological root resorption detected in periapical radiographies. The following exclusion criteria were used: excessive pulp bleeding until 5 minutes after removal; dark red colored pulp; little cut resistance; impossibility of restoration, presence of systemic illness and history of allergic reaction to the tested materials, latex or local anesthetics; and teeth with more than two thirds of radicular resorption. Based on the study of Moretti, et al.^6^ (2008), a sample size of 15 teeth *per* group was calculated to provide a power of 80% to detect statistical significance at the level of 5%.

### Randomization and blinding

Teeth included in this study were randomly allocated to one of three treatment groups using a computer-generated random number sequence. Each tooth was mapped to a single unique code until the end of the study, and randomization was concealed with the use of a sealed envelope. Based on the randomization, each tooth was allocated to one of the three experimental groups: MTA group, CH+saline group and CH+PEG group. Blinding of the outcome assessment and data analysis was achieved by using codes to designate each technique.

### Pulpotomy

Two investigators selected the teeth based on the inclusion criteria, but only one performed the pulpotomies. The investigators had been previously involved in several similar studies and used a standard pulpotomy technique. In all groups, caries removal was performed with a round bur in a slow-speed handpiece after local anesthesia with 2% mepivacaine with 1:100.000 epinephrine and rubber dam isolation. The pulp access cavities were performed with a round carbide bur. Full coronal pulp tissue (of appropriate consistency, bright- and red-colored and with cut resistance) was manually removed with an excavator, and the wound surface was irrigated with saline solution to remove debris and achieve pulp homeostasis. In the MTA group, a paste obtained by mixing gray MTA powder (Ângelus, Londrina, PR, Brazil) with distilled water at a 1:1 powder/liquid ratio, according to the manufacturer's specifications, was placed into the channel orifice in contact with the remaining dental pulp tissue. In the CH+saline group, the remaining pulp tissue was dressed with a paste obtained by mixing calcium hydroxide P.A. (Biodinâmica Química e Farmacêutica Ltda., Ibiporã, PR, Brazil) with sterile normal saline solution (0.9% sodium chloride) at a 1:1 powder/liquid ratio. In the CH+PEG group, the remaining pulp tissue was dressed with a paste obtained by mixing calcium hydroxide P.A. with PEG (MAPRIC - Produtos Farmacêuticos e Cosméticos Ltda., São Paulo, SP, Brazil) at a 1:1 powder/liquid ratio. In all groups, a 1-mm thick layer of material was used for capping, followed by another 1-mm thick of a layer of cement-cured calcium hydroxide (Biodinâmica Química e Farmacêutica Ltda., Ibiporã, PR, Brazil), employed as an intermediate base for the restoration with resin-modified glass-ionomer cement (RMGIC) (Vitremer; 3M ESPE, São Paulo, SP, Brazil).

### Clinical analysis and radiography

Periodic follow-up examinations were carried out 3, 6, and 12 months after treatment. At each follow-up appointment, two blinded and calibrated investigators performed clinical and periapical radiographic examinations of the pulpotomized teeth, as previously described.^6^ ^,^ ^11^ ^,^ ^13^ Inter- and intra-rater agreements were estimated as Kappa coefficients of 0.83 and 0.97, respectively. Patients were instructed on how to brush their teeth, and were encouraged to brush twice a day.

Standardized periapical or interproximal radiographs were taken with the use of conventional films (Sensitivity Insight films of the Kodak14 Mark, size #1) and holders, using an exposure time of 0.5 seconds.

Clinical success was defined as the lack of spontaneous pain, mobility, swelling, or fistula in the treated tooth. Lack of internal or external root resorption and furcation radiolucency were indicative of radiographic success. Data were tested by chi-squared analysis and a multiple comparison post-test, with statistical significance set at *p*<0.05.

## Results

**Figure 1:**
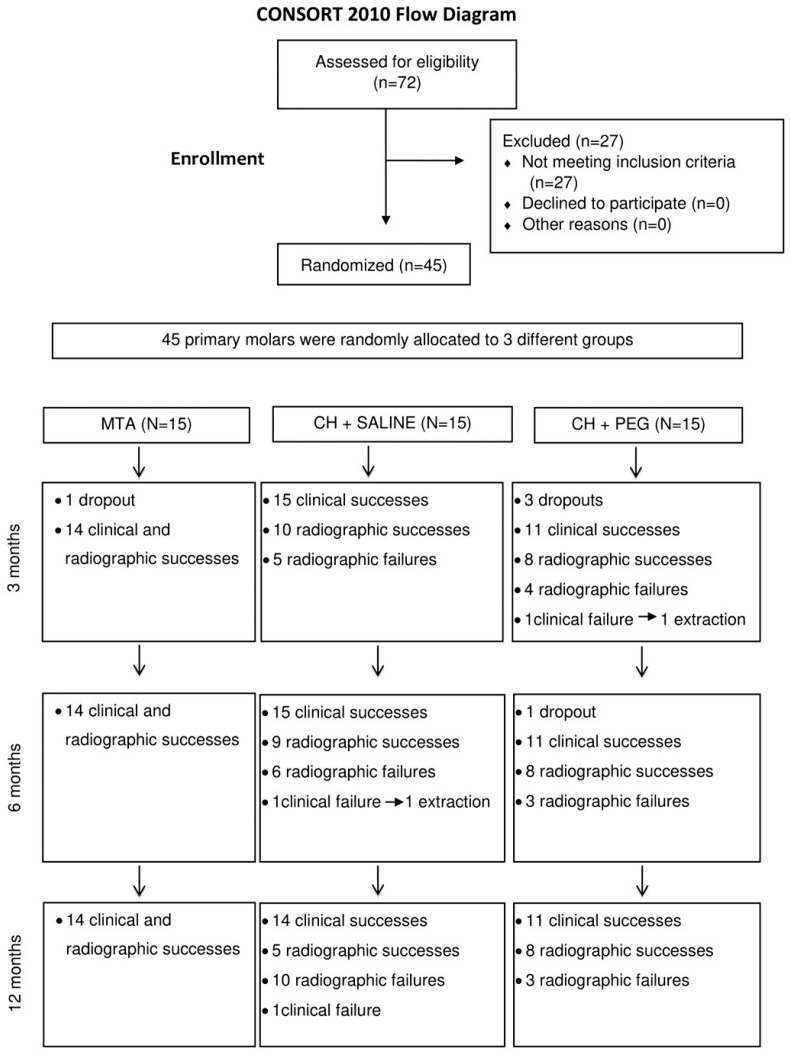
Flow of patients and pulpotomized teeth up to 12 months

Forty-five primary molars in children with mean age of 6 years and 5 months filled the inclusion criteria and were allocated to one of the three treatment groups (15 teeth per group) (Figure 1).

In all treatment groups, no tooth showed mobility, fistula, swelling or inflammation on the surrounding gingival tissue by clinical examination, at any follow-up appointments. In the MTA group, treatment was also successful by radiographic analysis in 14/14 teeth (100%). Thus, both clinical and radiographic analyses showed 100% treatment success using MTA, at all follow-up appointments (Figure 1; Table 1).

**Table 1:** Radiographic success (S) and failure (F) rates for the use of MTA, CH+saline and CH+PEG as capping agents in pulpotomies, at 3-, 6- and 12-month follow-up

Treatment	3 months		6 months		12 months	
	S	F	S	F	S	F
MTA	14 (100%)	0	14 (100%)	0	14 (100%)	0
CH + saline	10 (67%)	5 (33%)	9 (60%)	6 (40%)	5 (33%)	10 (67%)
CH + PEG	8 (75%)	3 (25%)	8 (73%)	3 (27%)	8 (73%)	3 (27%)

Despite the absence of clinical symptomatology indicative of treatment failure in the CH+saline and CH+PEG groups, we observed some radiographic evidence of treatment failure in these groups (Table 1). In the CH+saline group, internal resorption indicative of treatment failure was radiographically detected in up to 9/15 teeth (67%), at 12-month follow-up (Table 2). Inter-radicular bone destruction and furcation radiolucency, which also provide evidence of treatment failure, were observed in up to 5/15 teeth (36%), at 12-month follow-up (Table 3). Consequently, the final success rate of treatment in the CH+saline group was only 33% (5/15 teeth), after 12 months of follow-up (Table 1).

**Table 2:** Internal resorption radiographically observed for MTA, CH+saline and CH+PEG pulpotomies at 3-, 6-, and 12-month follow-up

Groups	3 months	6 months	12 months
MTA	0^b^	0^b^	0^b^
CH + saline	5 (33%)^a^	6 (40%)^a^	9 (67%)^a^
CH + PEG	2 (17%)^ab^	2 (18%)^ab^	2 (18%)^b^

* Different superscript letters mean significant differences between groups at 3, 6 and 12 months follow-up - chi-squared analysis and a multiple comparison post-test with 5%

**Table 3:** Inter-radicular bone destruction radiographically observed for MTA, CH+saline and CH+PEG pulpotomies at 3-, 6-, and 12-month follow-up

Groups	3 months	6 months	12 months
MTA	0^a^	0^b^	0^b^
CH + saline	3 (20%)^a^	4 (29%)^a^	5 (36%)^a^
CH + PEG	1 (8%)^a^	1 (9%)^ab^	1 (9%)^ab^

* Different superscript letters mean significant differences between groups at 3, 6 and 12 months follow-up - chi-squared analysis and a multiple comparison post-test with 5%

In the CH+PEG group, representing teeth capped with CH mixed with PEG, 2/12 teeth (17%), 2/11 (18%) and 2/11(18%) had internal resorption at the 3, 6 and 12 months of follow-up, respectively (Table 2), while one tooth also presented bone destruction and furcation radiolucency at all follow-up appointments (Table 3), being referred for endodontic treatment. The final radiographic success rate of the use of CH+PEG at 12-month follow-up was 73% (8/11 teeth).

The incidence of internal resorption in the CH+saline group was significantly higher than that in the MTA group, at all follow-up appointments (p=0.058; p= 0,019; p=0.0005). No statistically significant difference in the incidence of internal resorption was observed between the MTA group and the CH+PEG group throughout follow-up (Table 2).

**Figure 2:**
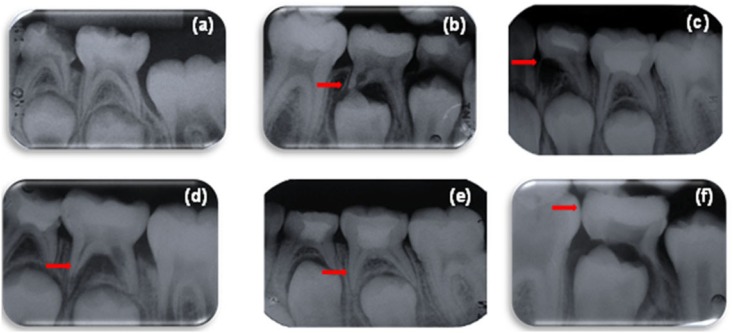
Sample radiographs representative of radiographic changes during follow-up. Arrows indicate the region of interest. (a) Preoperative radiograph; (b) Internal root resorption without perforation; (c) Internal root resorption with perforation; (d) Inter-radicular bone destructions; (e) Radiographic success; (f) Tooth too close to exfoliation

Regarding inter-radicular bone destruction, no statistically significant difference between groups was found at 3 months and 6 months (p=0.190; p=0.090). At 12 months of follow-up, the CH+saline group had an increased incidence of radiographic failure compared with the MTA group (p=0.026). Representative examples of radiographs taken during follow-up are shown in Figure 2.

## Discussion

In this study, we assessed the suitability of three different dressing agents (MTA, CH+saline and CH+PEG) for pulpotomy of primary teeth. This randomized clinical trial was carried out following a well established methodology employed previously regarding the inclusion and exclusion criteria, sample size, follow-up periods and clinical and radiographic criteria to determine the success or failure of pulpotomies.^6^ ^,^ ^11^ ^,^ ^13^

Pulpotomies with MTA had excellent outcomes over the entire follow-up period, while those employing CH-based capping materials had poorer outcomes, despite the fact that both materials have similar active chemical composition, which is directly influenced by its high pH. For both materials, the contact with vital tissues results in the dissociation of Ca^2+^ and OH^−^ ions. The Ca^2+^ ions react with carbon dioxide present in the tissue, resulting into calcite crystals and, thus, hard tissue deposition, while the OH^−^ ions have antimicrobial effect and cause pulp necrosis, which triggers tissue repair.^1^ ^,^ ^3^ ^,^ ^7^ The contrasting results with MTA and CH may be due to marginal differences in sealing ability as a result of distinct solubility or mechanical properties.^11^ ^,^ ^13^ ^,^ ^17^ The ability to maintain contact with the pulp tissue during the entire treatment period seems to be the main factor responsible for the excellent results with the use of MTA.^19^

Although CH+saline is commonly employed as a capping material for the pulp therapy of permanent teeth, this capping mixture was tested because results are contradictory regarding its clinical and radiographic success rates for the treatment of primary teeth.^6^ ^,^ ^12^ ^,^ ^13^ ^,^ ^20^ ^,^ ^21^ Moreover, CH-based materials tend to dissolve over time and leave a void underneath the restoration.^6^ ^,^ ^9^ To minimize this phenomenon, CH+PEG was also tested because the association of CH with a viscous vehicle could enhance the chemical and biological potential of the paste due to the slow release of Ca^2+^ and OH^−^ ions.^14^

Nelson-Filho, et al.^22^ (2005) reported 100% of clinical success, as well as 93% and 86.6% of radiographic success for MTA and CH with saline, respectively, after 6 months. Percinoto, Castro and Pinto^20^ (2006) reported that three out of forty-five teeth capped with CH failed after three months, two cases failed after six months and one failed after one year, while just two failures were found using MTA, after one year. Yildiz & Tosun^23^ (2014) concluded that the success rates of MTA and CH were 96.4% and 85%, respectively, after 30 months of follow-up. In contrast, after 36 months, Huth, et al.^13^ (2012) concluded that the radiographic success rate for pulpotomy with CH was 46% only. In our study, the success rate of CH+saline group decreased from 67% at 3 months follow-up to 33% at 12 months follow-up, in line with the results by Huth, et al.^13^ (2012), Mente, et al.^9^ (2014) and Moretti, et al.^6^ (2008). The progressive failure of pulpotomies using CH+saline may be due to the high solubility (through the radicular pulp tissue) of CH particles in the medium. When CH+PEG group was used, fewer failures (27%) were detected, which may be attributed to the incorporation of a viscous vehicle to the CH powder. The higher molecular weight of the viscous vehicle compared to the aqueous vehicles is likely to have reduced the dispersion of the CH powder through the pulp tissue, keeping the paste into direct contact with the tissue for longer.^14^

Internal resorption is the most frequent reason for failure following pulpotomy with CH in primary teeth, which indicates that, despite pulp vitality, a silent chronic inflammation develops after treatment with CH and remains undiagnosed, thus triggering odontoclast activity.^10^ ^,^ ^15^ Inappropriate operative techniques may also result in internal resorption if a thick blood clot remains or pulp misdiagnosis occurs.^20^ ^,^ ^23^ ^,^ ^24^ Thus, bleeding control after coronal pulp amputation may significantly influence the outcome of pulpotomies with CH.^23^ ^,^ ^24^ Some authors suggested that avoiding blood clot formation between the pulp tissue and the CH cap prevents the occurrence of internal resorption,^11^ however, this is technically difficult since the incision into vital tissue produces both haemorrhage and exudation.^17^ ^,^ ^25^ In the present study, we cannot attribute the occurrence of internal resorption to differences in the operative technique, since only one investigator performed all pulpotomies. In addition, the same method of bleeding control was employed in the three groups and no internal resorption was detected in the MTA group. It is worth mentioning that MTA has the advantage of hardening in the presence of moisture,^26^ which makes it suitable for use in areas where generating a dry environment is virtually impossible, such as pulp chambers.^12^ ^,^ ^6^

Following the guidelines of a prior study by Moretti, et al.^6^ (2015), we categorized internal resorption as radiographic failure, and the affected teeth were monitored during follow-up. We chose not to treat the affected teeth immediately because pathological findings in primary teeth may not require intervention, as primary teeth have limited survival and the pathology may not necessarily affect the permanent successor. However, most cases of internal resorption observed in CH-treated teeth in the present study developed into osseous changes whose clinical signs and symptoms were detected later on during follow-up, in agreement with that reported by Moretti, et al.^6^ (2015). Eventually, extraction of the affected teeth was necessary.

Dental pulp healing depends on several factors, including the presumed stimulatory effect of the capping agent, and the ability of both the capping and definitive restorative materials to seal the tooth restoration interface against immediate and long-term microleakage.^6^ ^,^ ^20^ In pulpotomies with CH, zinc oxide eugenol cement is commonly used before dental restoration.^10^ However, the fact that the CH paste dissolves easily could favor the contact of the healthy radicular pulp with the eugenol, which is considered aggressive, and could lead to failures.^6^ To avoid this effect, a resin-based CH cement was placed over the CH pastes as an intermediate base. This material has lower solubility and water sorption than the CH paste, increasing restoration longevity.^27^ Additionally, the CH cement is of low strength^28^ and is highly resistant to etchant,^29^ which is necessary for restorations with resin modified glass ionomer cement (RMGIC). Although stainless steel crowns provide optimal coronal seal,^6^ ^,^ ^13^ ^,^ ^17^ in the present study and in several others^6^ ^,^ ^10^ ^,^ ^11^ ^,^ ^24^ ^,^ ^30^ involving Class I and II cavities of primary molars in a high caries risk population, final restorations were performed with RMGIC. According to the guidelines of the American Association of Pediatric Dentistry (AAPD), there is strong evidence that RMGIC is efficient for Class I restorations, and expert opinion supports the use of RMGIC for Class II restorations in primary teeth,^31^ in line with a systematic review recommending the use of RMGIC in small to moderate sized Class II cavities.^32^ In support of the current recommendations, no restoration failures were observed in this study for any of the three groups throughout follow-up; thus, we can exclude restorative material as a discriminating factor between groups, in this study.

The limitations of this study are inherent to randomized clinical trials, where patients must return for follow-up and complete “blinding” may be challenging. In this study, 5 patients (one in the MTA group and four in the CH+PEG group) were lost at follow-up, because they were unwilling to travel from their hometown to report to the clinic. Moreover, it was impossible to blind the operator at the moment the material was inserted, since MTA and CH had different mixing methods. In addition, blinding the rater for the radiographic examination is not fail proof, as differences in radiopacity between materials can be identified by experienced examiners.

## Conclusion

The association of CH with PEG provided better results than that of CH with saline as a capping material for pulpotomy of primary teeth. However, both associations demonstrated clinical and radiographic results inferior to those of MTA.

## References

[1] Havale R, Anegundi RT, Indushekar K, Sudha P (2013). Clinical and radiographic evaluation of pulpotomies in primary molars with formocresol, glutaraldehyde and ferric sulfate. Oral Health Dent Manag.

[2] Junqueira MA, Cunha NN, Caixeta FF, Marques NC, Oliveira TM, Moretti AB (2018). Clinical, radiographic and histological evaluation of primary teeth pulpotomy using MTA and ferric sulfate. Braz Dent J..

[3] Yildirim C, Basak F, Akgun OM, Polat GG, Altun C (2016). Clinical and radiographic evaluation of the effectiveness of formocresol, mineral trioxide aggregate, Portland cement, and enamel matrix derivative in primary teeth pulpotomies: a two year follow-up. J Clin Pediatr Dent.

[4] Araújo LB, Cosme-Silva L, Fernandes AP, Oliveira TM, Cavalcanti BD, Gomes JE (2018). Effects of mineral trioxide aggregate, BiodentineTM and calcium hydroxide on viability, proliferation, migration and differentiation of stem cells from human exfoliated deciduous teeth. J Appl Oral Sci..

[5] Cosme-Silva L, Gomes-Filho JE, Benetti F, Dal-Fabbro R, Sakai VT, Cintra LTA (2018). Biocompatibility and immunohistochemical evaluation of a new calcium silicate-based cement, Bio-C Pulpo. Int Endod J..

[6] Moretti AB, Fornetti APC, Oliveira TM, Fornetti AP, Santos CF, Machado MA (2008). The effectiveness of mineral trioxide aggregate, calcium hydroxide and formocresol for pulpotomies in primary teeth. Int Endod J..

[7] Musale PK, Soni AS (2016). Clinical pulpotomy trial of Copaifera langsdorffii oil resin versus formocresol and white mineral trioxide aggregate in primary teeth. Pediatr Dent.

[8] Rajasekharan S, Martens LC, Vandenbulcke J, Jacquet W, Bottenberg P, Cauwels RG (2017). Efficacy of three different pulpotomy agents in primary molars: a randomized control trial. Int Endod J.

[9] Mente J, Hufnagel S, Leo M, Michel A, Gehrig H, Panagidis D (2014). Treatment outcome of mineral trioxide aggregate or calcium hydroxide direct pulp capping: long-term results. J Endod..

[10] Fernandes AP, Lourenço N, Teixeira Marques NC, Silveira-Moretti AB, Sakai VT, Cruvinel-Silva T (2015). Clinical and radiographic outcomes of the use of Low-Level Laser Therapy in vital pulp of primary teeth. Int J Paediatr Dent..

[11] Sakai VT, Moretti AB, Oliveira TM, Fornetti AP, Santos CF, Machado MA (2009). Pulpotomy of human primary molars with MTA and Portland cement: a randomised controlled trial. Br Dent J..

[12] Ozório JE, Carvalho LF, Oliveira DA, Sousa-Neto MD, Perez DE (2012). Standardized propolis extract and calcium hydroxide as pulpotomy agents in primary pig teeth. J Dent Child (Chic).

[13] Huth KC, Hajek-Al-Khatar N, Wolf P, Ilie N, Hickel R, Paschos E. (2012). Long-term effectiveness of four pulpotomy techniques: 3-year randomised controlled trial. Clin Oral Investig..

[14] Fava LR, Saunders WP (1999). Calcium hydroxide pastes: classification and clinical indications. Int Endod J.

[15] Oliveira TM, Sakai VT, Silva TC, Santos CF, Machado MA, Abdo RC (2008). Repair of furcal perforation treated with MTA in a primary molar tooth: 20-month follow-up. J Dent Child.

[16] Accorinte ML, Loguercio AD, Reis A, Carneiro E, Grande RH, Murata SS (2008). Response of human dental pulp capped with MTA and calcium hydroxide powder. Oper Dent..

[17] Waterhouse PJ, Nunn JH, Whitworth JM, Soames JV (2000). Primary molar pulp therapy - histological evaluation of failure. Int J Paediatr Dent.

[18] Cosme-Silva L, Carnevalli B, Sakai VT, Viola NV, Franco de Carvalho L, Franco de Carvalho EM (2016). Radicular perforation repair with mineral trioxide aggregate: a case report with 10-year follow-up. Open Dent J.

[19] Torabinejad M, Chivian N (1999). Clinical applications of mineral trioxide aggregate. J Endod.

[20] Percinoto C, Castro AM, Pinto LM (2006). Clinical and radiographic evaluation of pulpotomies employing calcium hydroxide and trioxide mineral aggregate. Gen Dent..

[21] Salako N, Joseph B, Ritwik P, Salonen J, John P, Junaid TA (2003). Comparison of bioactive glass, mineral trioxide aggregate, ferric sulfate and formocresol as pulpotomy agents in rat molars. Dent Traumatol.

[22] Nelson-Filho P, Venturini DP, Bezerra da Silva RA, Fiori-Junior M, Mori LB (2005). Mineral trioxide aggregate and calcium hidroxide utilized in pulpotomies in human primary teeth- clinical and radiographic study. J Health Sci Inst.

[23] Yildiz E, Tosun G (2014). Evaluation of formocresol, calcium hydroxide, ferric sulfate, and MTA primary molar pulpotomies. Eur J Dent.

[24] Oliveira TM, Moretti AB, Sakai VT, Lourenço N, Santos CF, Machado MA (2013). Clinical, radiographic and histologic analysis of the effects of pulp capping materials used in pulpotomies of human primary teeth. Eur Arch Paediatr Dent..

[25] Schröder U (1973). Effect of an extra-pulpal blood clot on healing following experimental pulpotomy and capping with calcium hydroxide. Odontol Revy.

[26] Nekoofar MH, Oloomi K, Sheykhrezae MS, Tabor R, Stone DF, Dummer PM (2010). An evaluation of the effect of blood and human serum on the surface microhardness and surface microstructure of mineral trioxide aggregate. Int Endod J.

[27] Francisconi LF, Freitas AP, Scaffa PM, Mondelli RF, Francisconi PA (2009). Water sorption and solubility of different calcium hydroxide cements. J Appl Oral Sci.

[28] Tam LE, Pulver E, McComb D, Smith DC (1989). Physical properties of calcium hydroxide and glass-ionomer base and lining materials. Dent Mater.

[29] Burke FJ, Watts DC (1989). Weight loss of three resin-based lining materials containing calcium following a phosphoric acid-etching and washing cycle. J Dent.

[30] Marques NC, Neto NL, Rodini CO, Fernandes AP, Sakai VT, Machado MA (2015). Low-level laser therapy as an alternative for pulpotomy in human primary teeth. Lasers Med Sci..

[31] American Academy of Pediatric Dentistry Reference Manual 2016 (2016). Guideline on Restorative Dentistry. Pediatr Dent..

[32] Chadwick BL, Evans DJ (2007). Restoration of class II cavities in primary molar teeth with conventional and resin modified glass ionomer cements: a systematic review of the literature. Eur Arch Paediatr Dent.

